# Alkaptonuria Presenting With Lumbar Disc Herniation: A Case Report

**DOI:** 10.7759/cureus.44395

**Published:** 2023-08-30

**Authors:** M L Bansal, Fazal Rehman T, Amlan Singh, Aayush Aryal

**Affiliations:** 1 Department of Spine Services, Indian Spinal Injuries Centre, New Delhi, IND

**Keywords:** black disc, ochronosis, spine, disc prolapse, alkaptonuria

## Abstract

Alkaptonuria is a rare autosomal recessive trait. Symptomatic lumbar disc herniation warranting surgical intervention is a rare scenario in alkaptonuria and only a few cases have been described in the literature. We present one such rare case of alkaptonuria in a 31-year-old female presenting with low back pain and left leg radiculopathy not relieved with conservative management. Roentgenograms of the lumbar spine revealed wafer-like disc calcifications and MRI showed a herniated disc at the L4-L5 level with deeply hypointense disc spaces in T2 suggestive of disc calcification and associated modic type 2 changes. During the surgery, the disc material removed was black in color, which raised a clinical suspicion of alkaptonuria. Postoperatively, the patient was re-examined and urine homogentisic acid was found to be raised. This, along with a histopathological examination, was diagnostic of alkaptonuria. The patient had excellent relief of symptoms postoperatively. In conclusion, if a ‘black disc’ is found during surgery, retrospective analysis and re-examination of patient clinical features and urine examination have to be done to diagnose alkaptonuria. While making a differential diagnosis of degenerative disc disease in patients with a calcified disc seen on radiography, a high index of suspicion for alkaptonuria has to be maintained.

## Introduction

Alkaptonuria (AKU) is a rare autosomal recessive trait [1] caused by a mutation in the Homogentisate 1.2-Dioxygenase (HGD) gene resulting in a lack of HGD enzyme function in the tyrosine and phenylalanine degradation pathways. The alkaptonuria gene (HGD) has been located at 3q21-q23 on the human chromosome 3q [2]. More than 90 unique HGD gene mutations have been reported around the globe [3,4]. Degeneration of cartilage, the intervertebral disc, and other connective tissues is caused by the accumulation of the pigment HGA and the resulting oxidation products. The degeneration in the spine most often affects the lumbar area [5-7].

The incidence of alkaptonuria is 0.001%. The large joints, including the spine, are the common areas involved in this condition. It usually presents with decreased flexibility and ankylosis of thoracolumbar segments, spondylosis, and canal stenosis. Lumbar disc herniation is a rare presenting characteristic of alkaptonuria, and only a few surgically treated cases of lumbar disc disease have been described in the literature [6-10]. In this case study, we present a rare case of alkaptonuria in which the patient had a lumbar disc herniation surgery. The disc material removed during his surgery was black in color, and his metabolic disease was identified retrospectively.

## Case presentation

A 31-year-old female presented to our walk-in clinic with complaints of chronic lower back pain and left lower limb radiating pain for three years and aggravated for six months. Leg pain was troubling her more than back pain and her walking distance was 10 minutes. No red flag signs were noted. There was nothing unusual about her family history or medical history. Spine examination revealed pain at the lower lumbar spine on flexion and a scoliotic lumbar curve was palpable with convexity to the left, which was getting corrected on forward bending. Neurological examination showed that dorsiflexion of the left toe was weak with a power of 4/5, and the straight leg raising test was positive on the left side.

**Figure 1:**
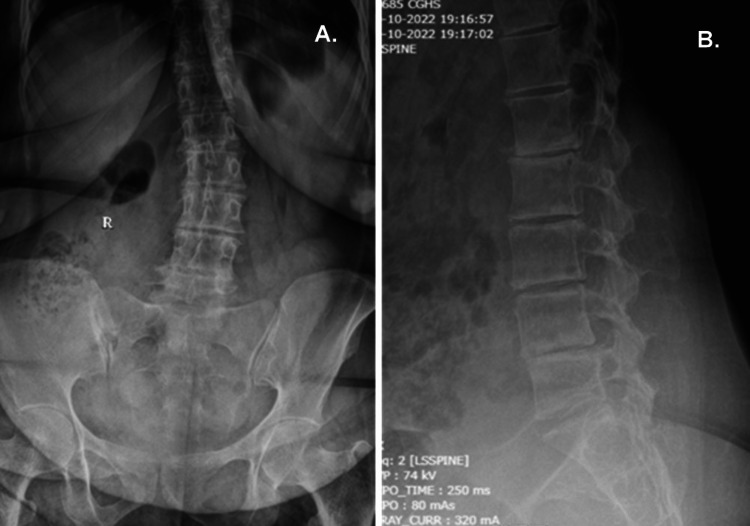
Spine radiographs lumbosacral anteroposterior (AP) view (A) and lateral view (B) showing degenerative changes in the spine

**Figure 2:**
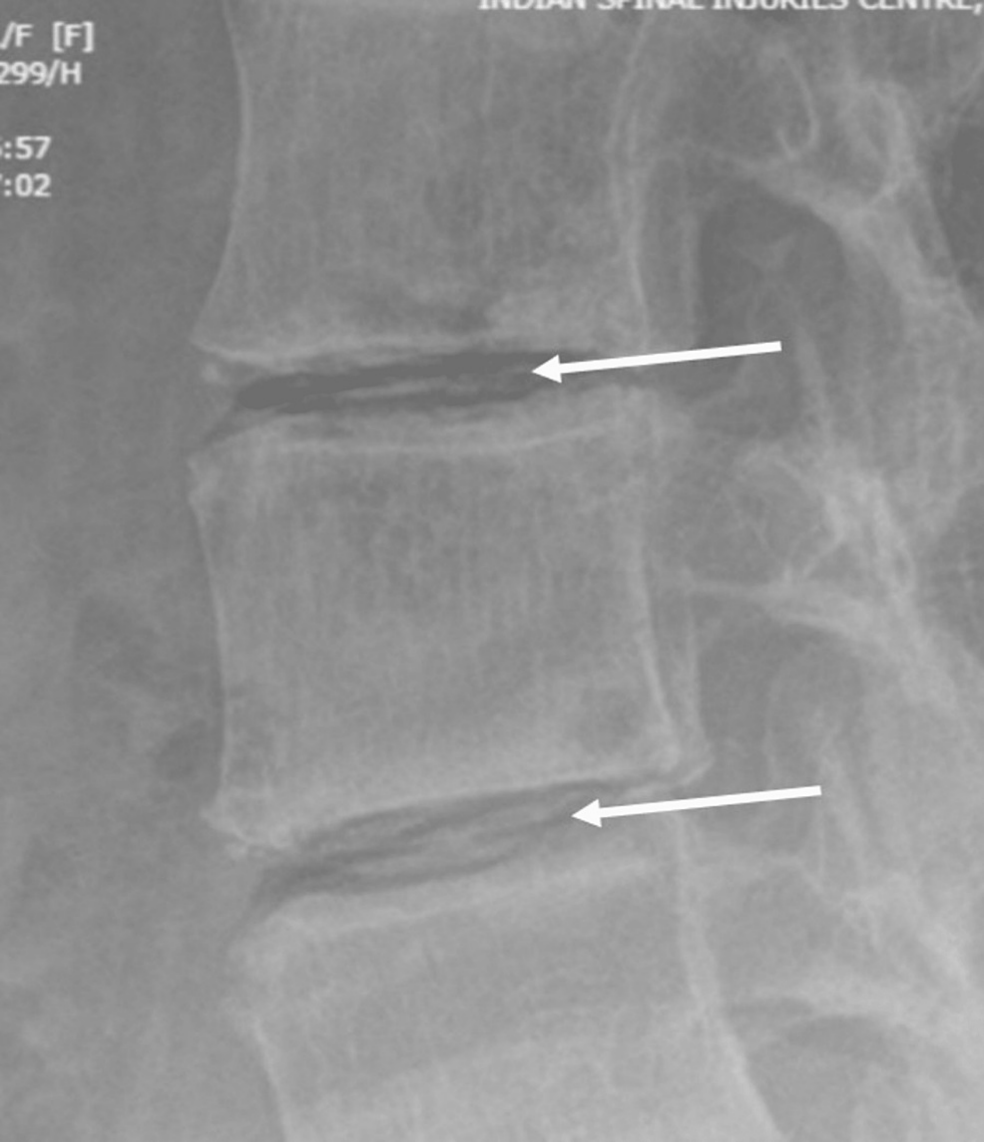
Showing calcification in the disc (white arrows)

**Figure 3:**
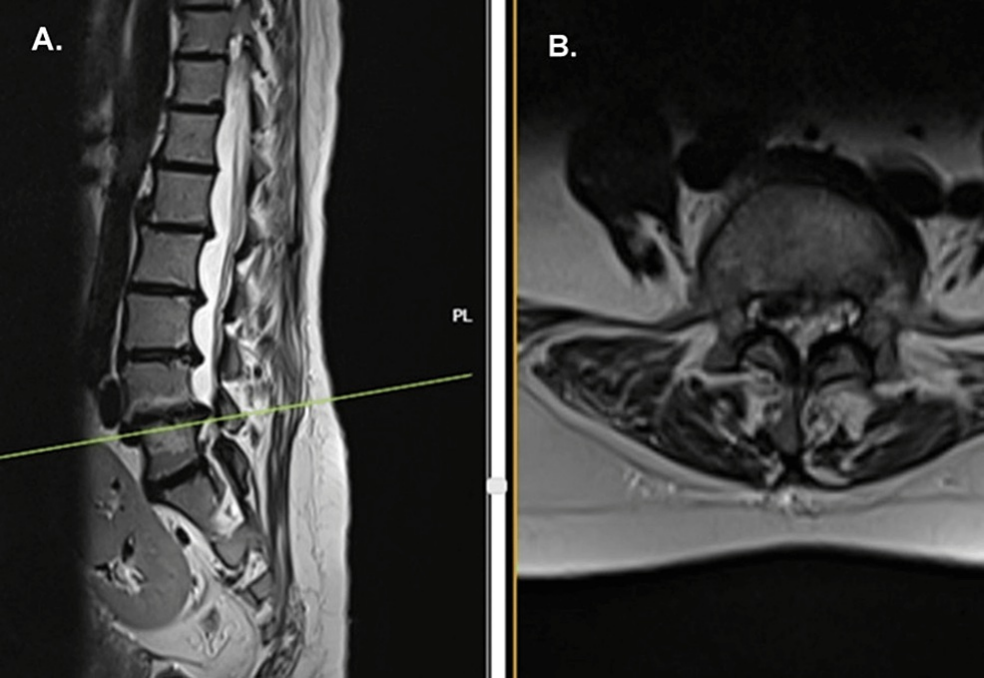
MRI sagittal view (A) and axial view (B) showing a prolapsed disc at the L4-L5 level extending to the right side and causing symptoms

Degenerative changes were seen on both the AP and lateral Roentgenograms of the lumbar spine (Figure 1). Intra-discal calcification was noted in multiple lumbar discs (Figure 2) along with end plate sclerosis, reduced bone density, and associated degenerative lumbar scoliosis. MRI scan revealed deeply hypointense disc spaces in T2 (Figure 3) and TIRM images due to calcification, which was confirmed in the corresponding levels on X-rays of the lumbosacral spine. There were Modic type 2 changes with end plate defects with Schmorl's node formation at multiple levels. There was a central and right paracentral disc prolapse at the L4-L5 level with inferior migration into the right lateral recess, causing her radicular leg pain.

**Figure 4:**
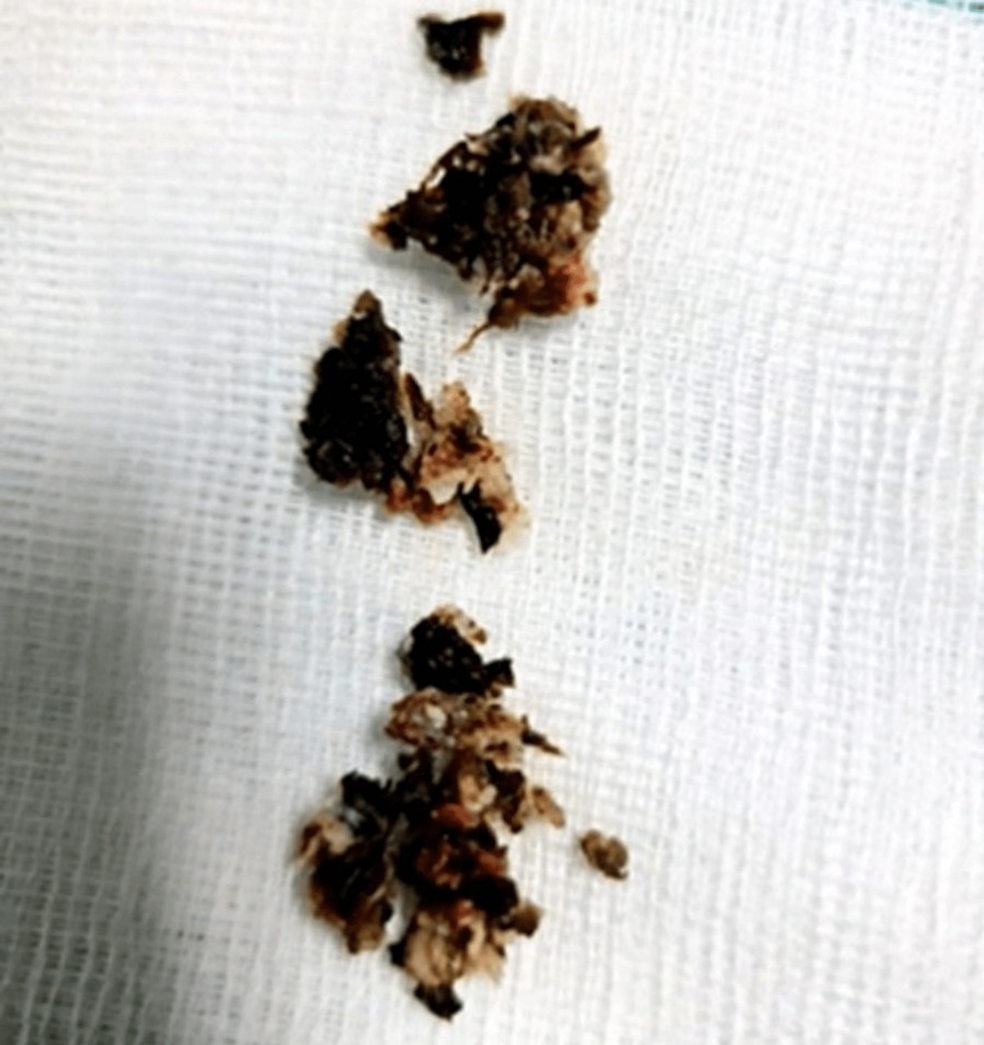
Macroscopic specimen of ‘black disc’ material obtained during surgery

As there was no relief in her symptoms even after six months of conservative management with analgesics, back exercises, activity modification, and physiotherapy, a decision was made to undergo surgical management. After counseling, she was admitted to the spine surgery unit. After calculating the Oswestry Disability Index (ODI) and visual analog scale (VAS) preoperatively, the patient underwent a discectomy with left-sided transforaminal lumbar interbody fusion (TLIF). Intraoperative findings involved extruded disc impinging on the thecal sac and L5 nerve root. The disc material removed was dark black (Figure 4). Disc material was sent for gram staining and bacterial cultures but reports came back negative. Pathological examinations revealed chromatin staining in chondrocyte cytoplasm suggestive of AKU. Postoperatively homogentisic acid was measured to be 1637% in a random sample of urine, which confirmed our diagnosis. After surgery, the patient no longer had any discomfort in her legs or lower back, and the postoperative period was uneventful.

Rheumatologist reference and monitoring were done for further management. Nitisinone, a diet low in protein, regular exercise, and pain medication were all used as part of the medical management strategy. Neurological examination, postoperative questionnaires (VAS and ODI), and six-month follow-up showed significant postoperative improvement.

## Discussion

Virchow, in 1866, developed the term 'ochronosis' to describe the disorder typified by the pigmentation of benzoquinone polymeric oxidation compounds from homogentisic acid (HGA) in the cartilage, ligaments, tendons, and intima of big blood vessels [11]. Many years later, in 1902, Albrecht and Zdareck reported its connection to alkaptonuria [11]. In 1915, Sodenberg described the arthritic condition known as 'osteitis deformans alkaptonuria' that affects the spine in people with ochronosis [12]. Lumbar stiffness, progressive loss of lordosis, and pronounced thoracic kyphosis are typical early symptoms [13]. Degenerative effects in the morphologic framework of connective tissue happen when homopolymeric oxidation products of HGA bind to collagen.

Alkaptonuric ochronosis is caused by the buildup of HGA and its oxidation products (such as benzoquinone acetic acid), causing blue-black pigmentation of connective tissue. A mild discoloration of the sclera or ears is the first sign of ochronosis, and it often appears in people between the ages of 20 and 30. However, only a minority of patients with alkaptonuria succumb to ochronosis or ochronotic arthropathy [13]. It is well-documented that a human liver can generate enough HGD to metabolize more than 1.5 kg of HGA daily [14]. As a result, for a patient to exhibit alkaptonuria symptoms, more than 99% of enzyme activity must be lost.

Clinically, dark urine or urine that becomes dark when left standing or exposed to an alkaline substance are common symptoms of alkaptonuria [4]. However, this darkening may occur many hours after urination, and many people never notice any change in the shade of their urine. Clinically, signs and symptoms often emerge in cases older than 30 while many remain asymptomatic in childhood and young adults [1]. In contrast to other studies, the re-examination of our patient did not reveal any discoloration of skin, sclera, or cartilage tissue. However, a darkening of urine was noted.

The three most common radiological findings in AKU were a narrowed disc space, the presence of osteophytes, and calcification, which, if present, causes the most rapid radiographic progression [15]. Though degenerative changes can happen in any part of the spine, the lumbar portion is most affected, followed by the dorsal and cervical regions. A calcified disc without calcification of ligaments, as in our case, is characteristic of this condition [7,16], but it is not diagnostic. The disc calcification manifests as oblong, opaque wafers between the vertebrae (Figure 2) [9,17]. It is very unusual for alkaptonuria to be diagnosed retrospectively by the presence of "black" disc material that was obtained during spine surgery. Postoperatively, such patients can be advised nitisinone, a low protein diet, and regular exercise. A close differential diagnosis of ochronosis is ankylosing spondylitis. However, the involvement of the intervertebral disc is the characteristic feature of the former, whereas, in ankylosing spondylitis, the spinal ligaments, facet joints, and sacroiliac joints are characteristically involved [18].

## Conclusions

Although alkaptonuric patients can be treated with supportive care, no proven effective prophylactic therapy is currently available. Corrective surgical techniques, such as discectomy or decompression with or without fusion, have been beneficial in this population of patients. In order to reduce morbidity, it's critical to identify problems early and treat them effectively, particularly in people who don't exhibit any other alkaptonuria symptoms.

Also, if a 'black disc' is found during surgery, retrospective analysis and re-examination of the patient's clinical features and urine examination have to be done to diagnose alkaptonuria. Therefore, as spine surgeons, while making differential diagnoses of degenerative disc disease, we should include metabolic disorders like alkaptonuria.
